# Efficacy of index of reactivity-liquid sublingual immunotherapy in allergic rhinoconjunctivitis: a systematic review and meta-analysis of randomized studies

**DOI:** 10.3389/falgy.2025.1597003

**Published:** 2025-06-05

**Authors:** Danilo Di Bona, Andrea Di Biase, Giovanni Paoletti, Rosanna Villani, Gaetano Serviddio, Josiane Cognet-Sicé, Silvia Scurati, Giorgio Walter Canonica

**Affiliations:** ^1^ Department of Medical and Surgical Sciences, University of Foggia, Foggia, Italy; ^2^ Department of Biomedical Sciences, Humanitas University, Pieve Emanuele, Italy; ^3^ Personalized Medicine, Asthma and Allergy, Humanitas Clinical and Research Center, IRCCS, Rozzano, Italy; ^4^ Integrated Health Care Department, Stallergenes Greer, Antony, France

**Keywords:** meta-analysis, randomized controlled trial, rhinitis, allergic, SLIT-liquid, sublingual immunotherapy, systematic review

## Abstract

**Introduction:**

Allergen immunotherapy (AIT) is a well-established treatment with demonstrated efficacy and safety. However, variability in study outcomes remains a challenge, driven by differences in patient characteristics, study designs, and treatment durations. Moreover, disparities in allergen composition and quality of AIT products across manufacturers contribute to significant heterogeneity, complicating the interpretation of efficacy and safety data. This meta-analysis focuses on assessing the efficacy and safety of a single manufacturer’s liquid sublingual immunotherapy (SLIT) for allergic rhinoconjunctivitis (ARC). By narrowing the scope to one specific product, this study seeks to reduce variability linked to product differences, aligning with recommendations from the World Allergy Organization to improve the reliability of meta-analytic findings.

**Methods:**

Randomized controlled trials (RCTs) on index of reactivity (IR) SLIT liquid formulations of various allergens were identified through comprehensive searches in electronic databases (MEDLINE, ISI Web of Science, the Cochrane Library, and ClinicalTrial.gov) up to December 2024, complemented by manual searches. Data on populations, treatments, and outcomes were extracted. Efficacy was evaluated by calculating the standardized mean difference (SMD) for symptoms and medication use. Subgroup analyses were performed by age, allergen type and sensitization status. Asthma comorbidity, dose and duration of SLIT were evaluated using meta-regression.

**Results:**

A total of 25 RCTs (1,830 patients) provided data on symptom scores (SS), and 19 RCTs (1,555 patients) reported on medication scores (MS). Analysis revealed that IR-SLIT-liquid was significantly more effective than placebo in reducing both SS (SMD: −0.30; 95% CI: −0.41 to −0.18; *P* < 0.0001) and MS (SMD: −0.51; 95% CI: −0.72 to −0.29; *P* < 0.0001). Efficacy outcomes were consistent regardless of factors such as age, allergen type (grass, house dust mites, trees, weeds), sensitization status, asthma presence, or cumulative dose, while longer treatment durations were associated with improved efficacy. No significant adverse events were reported.

**Discussion:**

This meta-analysis underscores the clinical effectiveness and safety of IR-SLIT-liquid, confirming its role as a reliable etiologic treatment for patients with ARC, for all allergens and age groups. The effect size is comparable to other immunotherapy options. The low rates of adverse events and treatment withdrawals highlight favorable tolerability and high level of patient adherence.

**Systematic Review Registration:**

https://inplasy.com/wp-content/uploads/2025/01/INPLASY-Protocol-7305.pdf, INPLASY 202510049

## Introduction

Allergic rhinoconjunctivitis (ARC) is one of the most widespread allergic conditions in developed nations, significantly affecting patients' daily lives (1). The symptoms often lead to disrupted sleep, reduced productivity at school or work, and limited social participation.

Allergen immunotherapy (AIT) is a proven treatment for allergies, as it targets the immune system's underlying response, offering a unique benefit over symptomatic treatments (1). AIT is commonly delivered through either subcutaneous (SCIT) or sublingual (SLIT) methods. Traditionally, SCIT has been the standard approach for treating ARC. However, in recent years, there has been a growing preference for SLIT, especially in Europe, where its use has risen to nearly the same frequency as SCIT (1).

Although numerous randomized controlled trials (RCTs) have demonstrated the efficacy of AIT in reducing symptoms and medication use, meta-analyses have highlighted considerable variability in outcomes (2–6). This variation can stem from differences in patient demographics, study designs, treatment regimens, and, importantly, the allergen products used, which can vary significantly across manufacturers. It is particularly worth noting that although some products are labelled with the same unit, e.g., the Index of Reactivity (IR), the definition of this unit may differ from product to product, resulting in discrepancies in allergenic activity (7, 8). These differences in formulation and quality may affect treatment efficacy, introducing additional heterogeneity into the data. To address this, the World Allergy Organization (WAO) and the European Academy of Allergy and Clinical Immunology (EAACI) advocate for product-specific meta-analyses to improve the consistency and reliability of results (9).

This article focuses on a product-specific meta-analysis of index of reactivity (IR) SLIT liquid formulations for ARC, comparing its efficacy against placebo across a range of common allergens, including grass pollen, house dust mite, tree and weed pollen extracts. The objective of this study is to quantitatively assess the clinical efficacy of IR-SLIT-liquid in reducing ARC symptoms and medication use, based on data from RCTs. Given the documented heterogeneity in previous meta-analyses, our hypothesis is that focusing on a single, standardized product will yield more consistent and robust evidence of clinical benefit compared to placebo.

## Methods

### Search strategy and selection criteria

This systematic review and meta-analysis were conducted according to PRISMA, GRADE, and Cochrane guidelines (10–12). This study was registered with the International Platform of Registered Systematic Review and Meta-analysis Protocols, INPLASY (registration number 202510049).

We performed a comprehensive search for published and unpublished RCTs on the efficacy of IR-SLIT liquid formulations for ARC in PubMed/MEDLINE, the Cochrane Library, ISI Web of Science, and ClinicalTrial.gov, up to December 20, 2024. No language restrictions were applied, and reference lists from relevant articles and reviews were manually checked for additional studies. We also asked the study sponsor to help provide a complete list of RCTs on IR-SLIT-liquid (Staloral®, Stallergenes Greer, Antony, France) with any allergen for ARC. A full list of the search terms is available in the protocol and the appendix (Supplementary Table S1).

Studies included in the analysis had to meet the following criteria: (1) adult and pediatric ARC patients, regardless of asthma status, with common allergens (grasses, house dust mites, trees, weeds); (2) treatment with IR-SLIT-liquid (Staloral®) for ARC; and (3) inclusion of relevant outcome measures such as symptom or medication scores. Reviews, discussion papers, non-research letters and editorials, animal studies, studies not employing double blind RCT designs, and studies not reporting necessary data were excluded.

### Data collection

Titles and abstracts were screened independently by two reviewers (AD, GP), followed by full-text review, data extraction, and risk of bias assessment using a pre-piloted form. Discrepancies were resolved by discussion with a third reviewer (RV). Study characteristics, patient populations, interventions, and outcomes were collected.

### Outcomes

Key outcomes were symptom severity (measured by symptom score, SS, or visual analog score, VAS), reduction in medication use (measured by medication score, MS), and safety (adverse events) (13).

### Data analysis

We conducted meta-analyses utilizing both fixed-effects and random-effects models, with a preference for the latter to account for anticipated variability across studies, including differences in protocols, durations, and populations (14). Continuous outcomes (e.g., SS, MS, VAS) measured on differing scales were combined using the standardized mean difference (SMD).

For studies examining outcomes over multiple pollen seasons, only data from the final year of treatment were included. When standard deviations (SDs) were not reported, we derived them using methods based on summary statistics (e.g., minimum, maximum, quartiles, median, or *p*-values) (15). In cases where standard errors (SEs) were provided, SDs were calculated using the formula: *SD* = *SE*√*n* (15). Missing means and SEs were estimated from graphs or obtained from the study sponsor.

The risk of bias (RoB) in RCTs was assessed with the Cochrane RoB 2 tool, which evaluates potential biases across five domains: randomization, adherence to interventions, outcome data completeness, measurement of outcomes, and selective reporting (16). Studies were rated as having a low or high risk of bias, or as raising some concerns. A study was categorized as low risk if no domains showed concerns, while a high-risk rating required substantial issues in one or more domains (16).

The certainty of evidence was appraised using the GRADE framework (11). Evidence was classified as high, moderate, low, or very low certainty based on confidence in the effect estimate. For instance, high-certainty evidence reflects strong confidence that the true effect is close to the estimate, while very low certainty suggests substantial uncertainty about the effect size.

To evaluate between-study heterogeneity, we employed the *χ*² test (*p*-threshold < 0.10) and *I*^2^ statistic, which quantifies the proportion of variability due to heterogeneity rather than chance (17). Potential sources of heterogeneity were examined through prespecified subgroup analyses, sensitivity analyses, and outlier detection using Baujat plots, which identify studies with disproportionate influence on heterogeneity and overall results (18). Meta-regressions further explored the relationship between outcomes and explanatory variables.

Sensitivity analyses included testing fixed-effects models, stratifying by data type (estimated vs. reported), study sample size, trial quality, and excluding duplicate data. Robustness was checked by systematically excluding individual studies to ensure no single study disproportionately influenced the results.

We assessed publication bias using funnel plots, Egger's regression test, and fail-safe calculations, which estimate the number of missing studies needed to overturn statistically significant results (19). A high fail-safe number provides confidence in the robustness of conclusions.

Summary of findings tables were generated using GRADEpro GDT software (20). Statistical analyses and meta-analyses were performed using R with the Metafor package, RevMan 5.0, and ProMeta 3.0 (21–23).

## Results

Our literature search retrieved 851 records. Following initial screening, 257 studies were reviewed in full, and 26 RCTs were finally included in the analysis (Supplementary Figure S1). Data for SS were reported in 25 studies, encompassing 1,830 patients (24–31, 33–49). Data for MS were available from 19 RCTs with a total of 1,555 participants (25–30, 33–37, 39, 40, 42–44, 47–49). The Sieber et al. study (32), which evaluated safety outcomes from the ECRIT trial reported by Ott et al. (26), provided data only for safety assessments and was excluded from the meta-analysis.

The characteristics of the included studies, comprising 25 trials for meta-analysis and the safety-focused study by Sieber et al., are summarized in Table 1. Most studies (*n* = 20) were conducted in Europe, with others carried out in Iran, Australia, South Africa, Taiwan, and Canada. Completion rates across trials averaged 83.1%. The risk of bias assessment identified 4 studies as high risk, 12 with some concerns, and 9 as low risk (Supplementary Figure S2). Sample sizes varied significantly, ranging from 15 participants in the smallest study (36) to 574 in the largest (47). Ten studies focused on pediatric populations, while 13 included only mono-sensitized patients. The proportion of participants with asthma varied widely, from 11.7%–100%. Treatment duration ranged from 4 months–36 months. The cumulative annual dose of AIT spanned from 4,500 IR–140,400 IR (Table 1).

**Table 1:** Patient and study characteristics.

Study, year Country	Patients	Male	Age, yr	Mono-/Poly-sensitized	Rhinitis	Asthma	Duration (months)	Maintenance Dose (IR)	Cumulative dose (IR)
	*N*	*N* (%)	mean ± SD (range)		(%)	*N* (%)			
Grass									
Sabbah et al. (24)	AIT 29 → 29	31 (53.4)	23 ± 10 (13–43)	Poly-	100	n.r.	4	100	4,500
France	C 29 → 29		27 ± 12 (13–51)			n.r.			
Clavel et al. (25)	AIT 62 → 62	71 (59.2)	29 ± 13 (9–55)	Poly-	100	10 (16)	6	300	40,700
France	C 58 → 58		26 ± 12 (8–55)			16 (27.6)			
Ott et al. (26)	AIT 123 → 99	71 (38.8)	33.2 ± 11.0	Poly-	100	14 (14.1)	36 (3 × 3)	300	66,000 (22,000/yr)
Germany	C 60 → 46		33.7 ± 9.1			5 (10.9)			
Stelmach et al. (27)	AIT 25 → 20	22 (44)	9.1 ± 2.4 (6–17)	Mono-	n.r.	20 (100)	24 (2 × 6)	120	43,800 (21,900/yr)
Poland	C 25 → 15		8.5 ± 2.8		n.r.	15 (100)			
Kałuzińska et al. (28)	AIT 15 → 13	19 (63.3)	8.3 ± 3.3 (6–18)	Mono-	100	4 (30)	24 (2 × 6)	120	43,800 (21,900 yr)
Poland	C 15 → 12		8.1 ± 3.3			3 (25)			
Stelmach et al. (29)	Pre-co 17 → 17	36 (66.7)	8.3 (5–17)	Mono-	100	6 (35)	24	240	87,600 (43,800/yr)
Poland	Cont. 19 → 19		10.1 (3–16)			5 (26)	(6 × 2)		
	C 18 → 18		8.1 (4–15)			5 (18)	(12 × 2)		
Bozek et al. (30)	AIT 41 → 38	41 (52.6)	63.18 ± 3.12	Mono-	100	3 (7.32)	36 (4 × 3)	240	66,000 (22,000/yr)
Poland	C 37 → 34		64.13 ± 2.92			2 (5.4)			
Kralimarkova et al. (31)	AIT 28 → 21	33 (58.9)	30.3 ± 12.6	Mono-	100	10 (36)	5	300	45,000 (108 000/yr)
Bulgaria	C 28 → 24		30 ± 12.5			10 (36)			
Sieber et al. (32)	AIT 142 → 132	n.r.	(7.9–64.7)	n.r.	100	n.r.	36 (4 × 3)	300	66 000 (22,000/yr)
Germany	C 67 → 63					n.r.			
Rye Grass									
Ahmadiafshar et al. (33)	AIT 12 → 10	5 (25)	8.13 ± 2.5	Mono-	100	n.r.	6	n.r.	n.r.
Iran	C 12 → 10		9.14 ± 6.4			n.r.			
HDM									
Mungan et al. (34)	AIT 15 → 15	2 (13.3)	31.67 ± 7.28	n.r.	100	86	12	100	11,316
Turkey	C 11 → 11		(18–41)						
Guez et al. (35)	AIT 36 → 25	14 (38.8)	29.6 ± 12.4	Poly-	100	n.r.	24	300	90,000 (45,000/yr)
France	C 36 → 14		(12–51)						
Bahceciler et al. (36)	AIT 8 → 8	4 (50)	12.4	Mono-	100	100	6	100	7,000 (14,000/yr)
Turkey	C 7 → 7		(7.8–18)						
Tseng et al. (37)	AIT 30 → 28	22 (73)	9.7 ± 3.3	Mono-	100	0	6	300	37,312 (74,424/yr)
Taiwan	C 33 → 31		9.7 ± 3.0						
O'Hehir et al. (38)	AIT 15 → 13	3 (33.3)	28.5 ± 8.2	Poly-	100	77.7	12 (DB) +	300	85,621 (42,810/yr)
Australia	C 15 → 14		37.6 ± 11.1				12 open		
Aydogan et al. (39)	AIT 9 → 7	6 (85)	8.1 ± 2.2	Mono-	100	0	12	300	44,500
Turkey	C 9 → 9		7.3 ± 2.3						
Bozek et al. (40)	AIT 51 → 47	23 (45)	65.8 ± 4.9	Mono-	100	11.7	36	240	421,200 (140,400/yr)
Poland	C 57 → 48		66.7 ± 3.8						
Potter et al. (41)	AIT 39 → 32	14 (35.9)	33.7	Poly-	100	n.r.	24 (3 days/wk)	300	96,600 (48,300/yr)
South Africa	C 21 → 16		31.4						
Trees									
Di Rienzo et al. (42)	AIT 19 → 18	20 (58.8)	33.8 ± 9.5	No perennial allergens	100	n.r.	4	300	36,000
Italy (Cypress)	C 15 → 14								
Khinchi et al. (43)	AIT 23 → 14	23 (62)	30 (20–58)	No perennial allergens	100	n.r.	12	49.2 *μ*g every other day	11 mg
Denmark (Birch)	C 24 → 15								
Vervloet et al. (44)	AIT 38 → 36	39 (51.3)	39 (19–60)	No perennial allergens	100	13.1	4	300	36,000
France (Cypress)	C 38 → 34								
Voltolini et al.(45)	AIT 14 → 13	10 (41.7)	41.8 ± 8	Mono-	100	100	24 (4 × 2)	300	72,000
Italy (Birch)	C 10 → 9								
Vourdas et al. (46)	AIT 34 → 33	49 (74.2)	12 (7–17)	Poly- (85%)	100	88	24 (6 × 2)	300	108,000
Greece (Olive)	C 32 → 31								
Worm et al. (47)	AIT 284 → 247	258 (48.1)	37.5 ± 11.1	Poly- (72%)	100	25	24 (5 × 2)	300	90,000
Germany (Birch)	AIT 290 → 253								
Weeds									
Bowen et al. (48)	AIT 43 → 37	45 (59.2)	36.3 (14–58)	Mono-	100	20	4	300	17,450
Canada (Ragweed)	C 40 → 39								
La Rosa et al. (49)	AIT 20 → 16	25 (59.5)	10 (6–14)	Mono- (75.6)	100	n.r.	24	300	150,000 (75,000/yr)
Italy (Parietaria)	C 21 → 17								

N, number; SD, standard deviation; IR, index of reactivity; AIT, allergen immunotherapy; C, controls; →, number of patients from enrolment to the observation time-point; n.r., not reported; yr, year; wk, week; DB, double-blind.

**Figure 1:**
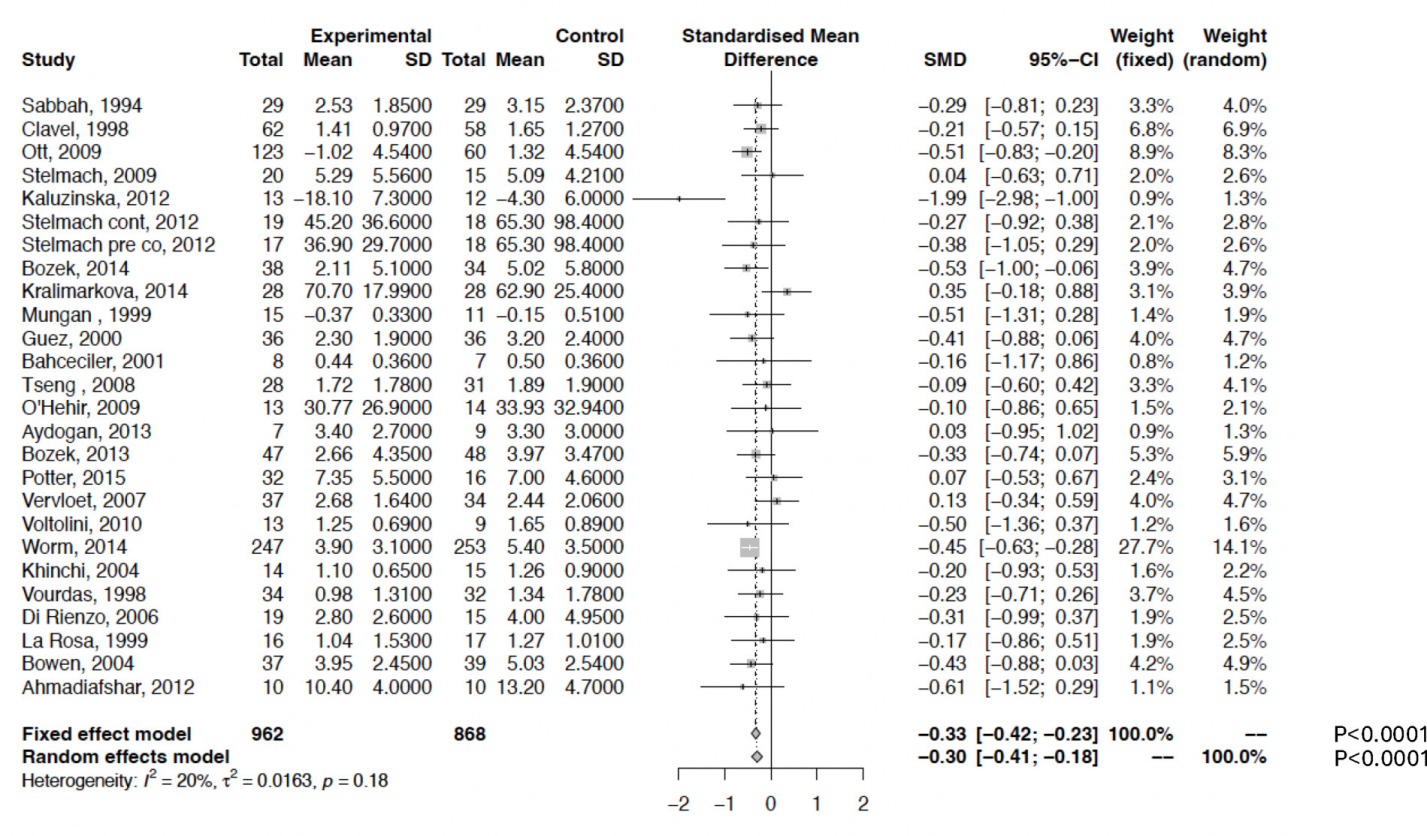
Meta-analysis of 25 RCTs of IR-SLIT-liquid vs. placebo for allergic rhinoconjunctivitis. The SMD and 95% CI for the effect of treatment on symptom score (SS) are plotted on the graph.

The impact of IR-SLIT-liquid on SS is illustrated in Figure 1. The three-arm trial reported by Stelmach et al. in 2012 (29) was treated as two separate studies due to the inclusion of two active treatment arms against a shared placebo group, leading to duplication of the placebo arm. A sensitivity analysis adjusting for this duplication by halving the placebo group size revealed no substantial differences in the results (Supplementary Table S2). The pooled SMD for treatment effects was −0.30 (95% CI, −0.41 to −0.18; *P* < 0.0001), indicating a significant benefit of SLIT compared to placebo. Results from the fixed-effects model were similar. Low heterogeneity was observed (Q = 0.37; df = 25; *P* = 0.22; *I*^2^ = 20%) but decreased to 0% after excluding three outlier studies (28, 31, 44) (Supplementary Figure S3B, Supplementary Table S2). These outliers were classified as low- or medium-quality studies (Supplementary Figure S2).

Visual inspection of funnel plots and Egger's test indicated no significant publication bias (Supplementary Figure S3A). The fail-safe number (*n* = 199) further supported the robustness of the results.

**Figure 2:**
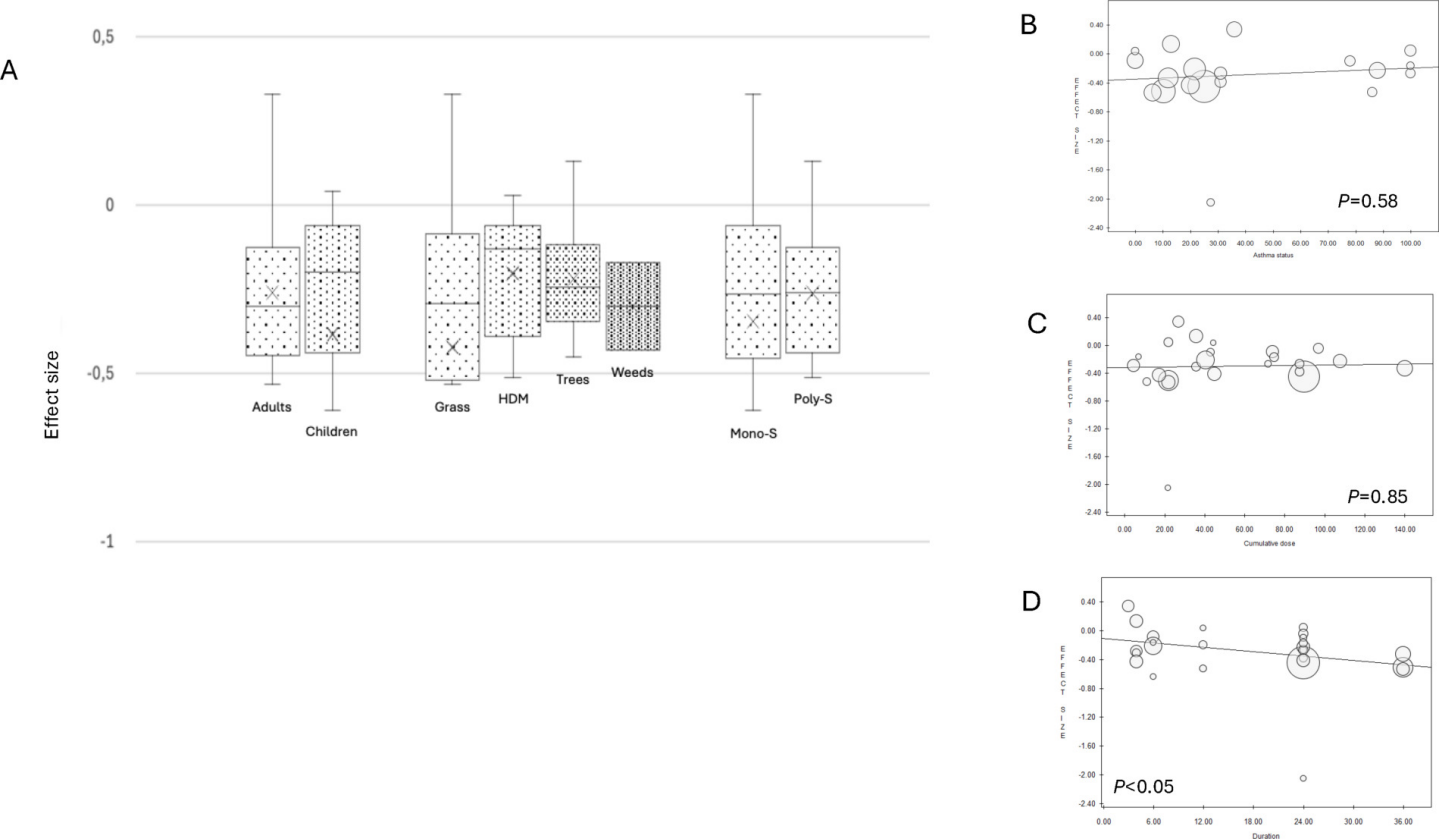
Subgroup analysis of SS. Box plots include middle 50% of data. Horizontal bars inside boxes represent SMD; lines to whiskers extend most extreme data points, which are no more than 1.5 times interquartile range from box **(A)** Meta-regression analyses of SS for efficacy of IR-SLIT-liquid depending on asthma status **(B)**, cumulative dose administered per year **(C)**, treatment duration **(D)**.

Subgroup analyses by age, allergen type, and sensitization status showed no significant differences across subgroups (Figure 2A). Meta-regressions indicated no substantial effect based on asthma status or cumulative annual AIT dose (Figures 2B,C), but treatment duration was positively associated with improved outcomes (Figure 2D).

**Figure 3:**
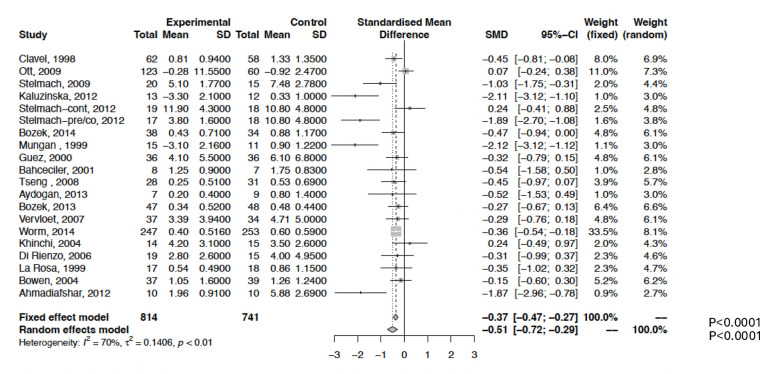
Meta-analysis of 20 RCTs of IR-SLIT-liquid vs. placebo for allergic rhinoconjunctivitis. The SMD and 95% CI for the effect of treatment on medication score (MS) are plotted on the graph.

Figure 3 presents data on MS outcomes. The pooled SMD was −0.51 (95% CI, −0.72 to −0.29; *P* < 0.0001) with considerable heterogeneity (*I*^2^ = 70%). However, excluding four influential studies (26, 28, 29, 34) eliminated heterogeneity (*I*^2^ = 0%) without altering the overall results (Supplementary Figure S4B, Supplementary Table S2). No evidence of publication bias was detected (Supplementary Figure S4A).

**Figure 4:**
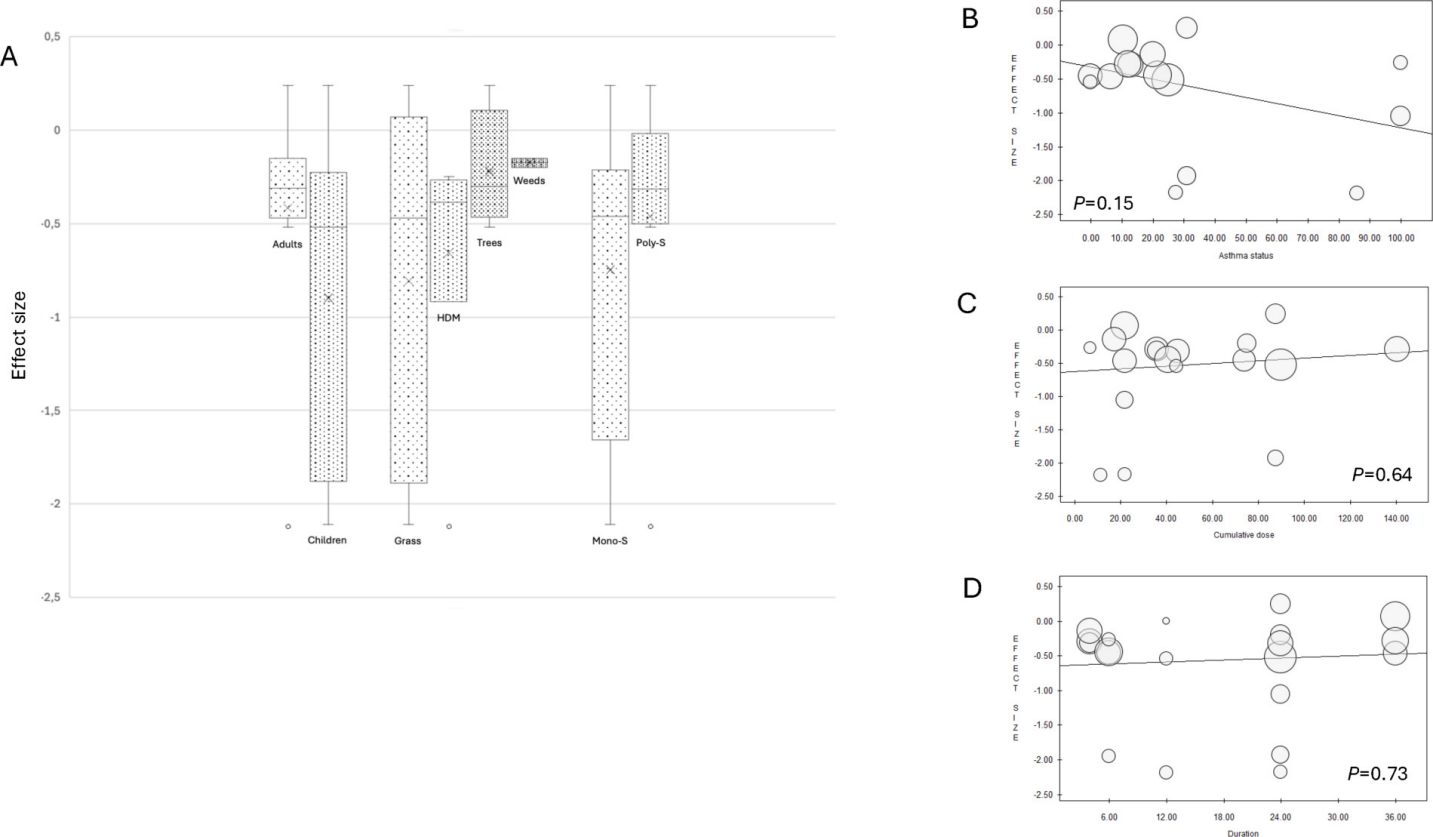
Subgroup analysis of MS. Box plots include middle 50% of data. Horizontal bars inside boxes represent SMD; lines to whiskers extend most extreme data points, which are no more than 1.5 times interquartile range from box **(A)** Meta-regression analyses of MS for efficacy of IR-SLIT-liquid depending on asthma status **(B)**, cumulative dose administered per year **(C)**, treatment duration **(D)**.

Subgroup analyses for MS revealed no significant differences by age, allergen, or sensitization status (Figure 4A). Meta-regressions similarly found no significant associations with asthma status, cumulative AIT dose, or treatment duration (Figures 4B–D).

Sensitivity analyses across various parameters—including estimated vs. reported data, study quality, sample size (above or below the median of 56 participants), and exclusion of influential studies or those with duplicate controls—confirmed the robustness of findings, particularly for SS (Supplementary Table S2).

The overall certainty of evidence was rated as moderate for both SS and MS outcomes (Supplementary Table S3).

Adverse events (AEs) data were available for 1,068 SLIT patients and 948 placebo patients (Table 2). AEs were reported by 44.7% of SLIT patients (*n* = 478) and 33.1% of placebo patients (*n* = 313), the difference between both groups being statistically significant (Table 2). Treatment discontinuation rate due to AEs was slightly higher in the SLIT group (3.9%) compared to the placebo group (1.9%; *P* < 0.05). Conversely, discontinuation for reasons unrelated to AEs was more common in the placebo group (*P* < 0.01) (Table 2).

**Table 2:** Adverse events.

Study	Patients, *n*		Patients with AE, *n* (%)		Patients discontinuing for reason other than AE, *n* (%)		Patients discontinuing for AE, *n* (%)	
	SLIT	Placebo	SLIT	Placebo	SLIT	Placebo	SLIT	Placebo
Sabbah et al. (24)	29	29	10 (34.5)	7 (24.1)	0	0	0	0
Clavel et al. (25)	62	58	18 (29.0)	10 (17.2)	20		0	0
Ott et al. (26)/Sieber et al. (32)	142	67	98 (69.0)	42 (62.7)	n.r.	n.r.	10 (7.0)	4 (6.0)
Stelmach et al. (27)	25	25	n.r.	n.r.	5 (20.0)	10 (40.0)	0	0
Kaluzinska et al. (28)	15	15	n.r.	n.r.	2 (13.3)	3 (20.0)	0	0
Stelmach et al. (29)	Pre-co 17	18	8 (47.1)	8 (44.4)	3 (17.6)	2 (11.1)	0	0
	Cont. 19		6 (31.6)		1 (5.3)			
Bozek et al. (30)	41	37	5 (12.2)	0	3 (7.3)	3 (9.7)	0	0
Kralimarkova et al. (31)	28	28	n.r.	n.r.	7 (25.0)	4 (14.3)	0	0
Ahmadiasfar et al. (33)	12	12	n.r.	n.r.	2 (16.6)	2 (16.6)	0	0
Mungan et al. (34)	15	11	2	n.r.	0	0	0	0
Guez et al. (35)	36	36	2 (5.5)	1 (2.8)	11 (30.5)	22 (61.1)	0	0
Bahceciler et al. (36)	8	7	0	0	0	0	0	0
Tseng et al. (37)	30	33	19 (63.3)	7 (21.2)	2 (6.7)	2 (6.1)	n.r.	n.r.
O'Hehir et al. (38)	15	15	9 (60.0)	2 (13.3)	2 (13.3)	1 (6.7)	0	0
Aydogan et al. (39)	9	9	1 (11.1)	0	1 (11.1)	0	1 (11.1)	0
Bozek et al. (40)	51	57	3 (5.6)	1 (1.7)	4 (7.8)	9 (15.8)	0	0
Potter et al. (41)	39	21	n.r.	n.r.	7 (17.9)	5 (23.8)	0	0
Di Rienzo et al. (42)	19	15	7 (36.8)	3 (20.0)	1 (5.3)	1 (6.7)	0	0
Khinchi et al. (43)	23	24	15 (65.2)	11 (45.8)	6 (26.1)	8 (33.3)	3 (13.0)	1 (4.2)
Vervloet et al. (44)	38	38	5 (13.2)	7 (18.4)	1 (2.6)	4 (10.6)	1 (2.6)	0
Voltolini et al. (45)	14	10	10 (76.9)	4 (44.4)	1 (76.9)	1 (11.1)	0	0
Vourdas et al. (46)	34	32	8 (23.5)	2 (6.4)	1 (2.9)	1 (3.1)	0	0
Worm et al. (47)	284	290	200 (70.7)	185 (63.8)	23 (8.1)	24 (8.3)	17 (6.0)	12 (4.1)
Bowen et al. (48)	43	40	30 (69.8)	16 (40.0)	9 (20.9)	11 (27.5)	6 (13.9)	0
La Rosa et al. (49)	20	21	12 (60.0)	7 (33.3)	1 (4.8)	3 (14.2)	4 (20.0)	1 (4.7)
TOTAL	1,068	948	478^a^ (44.7)	313^a^ (33.1)	93^b^ (8.7)	116^b^ (12.2)	42^c^ (3.9)	18^c^ (1.9)

AE, adverse events; *n*, number; Pre-co, pre-coseasonal; Cont., continuous; n.r., not reported.

^a^ *X^2^* = 12.2; *P* < 0.01.

^b^ *X^2^* = 1.45; *P* = 0.02.

^c^ *X^2^* = 2.99; *P* = 0.009.

## Discussion

This meta-analysis, encompassing data from 25 RCTs and over 1,800 patients with ARC caused by various allergens treated with IR-SLIT-liquid, demonstrates that this therapy effectively reduces both symptoms and the reliance on rescue medications without raising significant safety concerns. These findings align with previous studies supporting the efficacy and safety of SLIT in individuals with ARC, with or without coexisting mild to moderate asthma (4–6). The observed effect size is consistent with outcomes reported in previous meta-analyses combining not product-specific SLIT liquid formulations and/or SLIT tablets (2, 3).

Contrary to earlier reports pooling data from various SCIT and SLIT products, which suggested greater efficacy for house dust mite (HDM) immunotherapy compared to seasonal allergens, our findings demonstrate a consistent therapeutic benefit of IR-SLIT-liquid across a diverse range of allergens, including HDM, grasses, trees, and weeds (50). Adhering to the WAO and EAACI recommendations to focus on a single manufacturer's product minimized, at least for SS, variability related to product quality, resulting in more reliable and cohesive outcomes compared to broader analyses which were hindered by high heterogeneity (9).

The most recent RCTs (26, 47, 51) have demonstrated a favorable benefit-risk balance of IR-SLIT-liquid at the daily dose of 300 IR, which is the dose recommended in the product information. Nevertheless, meta-regression analysis of cumulative yearly dosage found no significant variation in efficacy across studies using different dose levels (Figures 2C,4C). This indicates that the dosage of the SLIT liquid formulation can be safely adjusted downward or upward, depending on the patients' profile and their response to the treatment, either to manage adverse events without compromising effectiveness or to enhance the latter, as is observed in real-life practice (52). Such flexibility allows treatments to be tailored to individual patient needs and preferences, promoting adherence and facilitating the recommended minimum treatment duration of three years (13), which is associated with improved outcomes. A recent real-world study using data from the French National Health Data System (SNDS), which encompasses 98.8% of the French population, demonstrated the effectiveness of IR-SLIT-liquid in reducing the risk of asthma onset and progression (53). Despite focusing on a different outcome, this study involved patients treated for at least 2 years in real-life conditions with significant variability in adherence and dosage. These findings further support the conclusion that achieving a specific cumulative dose is not critical for treatment efficacy, making the product more adaptable to patients' needs.

In contrast, SS meta-regression analysis by treatment duration revealed a positive association with the efficacy of IR-SLIT-liquid (Figure 2D). These findings highlight the importance of adhering to the recommended treatment duration, as longer treatment periods are associated with improved clinical outcomes, even with dosage variations. Specifically, the results complement those of some RCTs, which highlight that SLIT is particularly effective in patients who maintain treatment for at least 36 months (54). This extended duration not only aligns with current clinical guidelines but also reinforces the idea that sustained therapy is crucial for achieving optimal therapeutic benefit. These benefits refer exclusively to the on-treatment effect, as we did not report on long-term outcomes after discontinuation or on other potential AIT effects, such as the prevention of asthma or the occurrence of new sensitizations, due to the lack of available data on these endpoints in the included RCTs.

Subgroup analyses revealed that the efficacy of IR-SLIT-liquid was not influenced by age, with consistent outcomes observed in both adult and pediatric subgroups (Figures 2A,4A). When stratified by sensitization status, no significant differences were detected between mono-sensitized and poly-sensitized patients, even though some studies have suggested higher efficacy in mono-sensitized individuals for both SS and MS outcomes (Figures 2A,4A). Similarly, no variations in the efficacy of IR-SLIT-liquid were noted based on asthma prevalence (Figures 2B,4B), suggesting that asthma does not significantly impact patients' perception of ARC symptoms.

However, we acknowledge that certain unmeasured variables—including environmental exposure and concomitant medication—could not be systematically assessed due to limited reporting across the included studies.

Adverse event (AE) reporting varied widely across studies, with some not providing detailed information on this aspect. Overall, a higher number of patients in the SLIT group reported AEs compared to placebo (44.7% vs. 33.1%, respectively; *P* < 0.01). Only a small number of patients discontinued treatment, even in long-term trials, and withdrawal rates due to AEs were comparable between SLIT and placebo groups (Table 2). These findings highlight the good tolerability of the treatment. Furthermore, no cases of anaphylaxis were reported, underscoring its safety profile.

## Strengths and limitations

Focusing on a specific product significantly reduced heterogeneity, at least in SS, and led to consistent estimates between random- and fixed-effects models. This consistency strengthens confidence in the conclusion that the product is effective. Additionally, the low risk of publication bias, along with findings from sensitivity analyses, supports the robustness of the results.

However, a key limitation of this analysis is the small sample size in most of the included studies (median sample size: 56 patients), which likely contributes to inconsistencies across individual studies, as small studies are more prone to report better results than larger studies. This pattern was observed for MS only, but not for SS (Supplementary Table S2). Nonetheless, these factors reduced the certainty of evidence to moderate for both SS and MS (Supplementary Table S3). Another limitation of this meta-analysis is that the sources of heterogeneity could not be fully explained, despite our efforts to assess the role of various clinical baseline characteristics. Unfortunately, certain unmeasured variables—including environmental exposure and concomitant medication—could not be systematically assessed due to limited reporting across the included studies. Nevertheless, the IR-SLIT-liquid has shown beneficial effects in real-life studies with larger populations, strengthening its evidence for the causal treatment of patients with respiratory allergies (53, 55–57).

## Conclusions

This meta-analysis confirms that IR-SLIT-liquid is effective in improving rhinoconjunctivitis symptoms and reducing the need for symptomatic medications compared to placebo. The findings are consistent across various allergens, suggesting that the differences in outcomes reported with different allergens in other studies may be due to variations in product quality and standardization. Treatment efficacy is not affected by factors such as bronchial asthma, patient age, or cumulative dose. However, it is linked to treatment duration, indicating that reducing the dose to manage side effects does not compromise overall effectiveness, provided the treatment is continued over time. Furthermore, the effect size is comparable to other immunotherapy options. The low rates of adverse events and treatment withdrawals highlight favorable tolerability and high level of patient adherence. Overall, IR-SLIT-liquid could be considered a reliable etiologic treatment for patients with ARC, for all allergens and age groups.

## Data Availability

The original contributions presented in the study are included in the article/Supplementary Material, further inquiries can be directed to the corresponding author.

## References

[1] Bousquet J Schünemann HJ Zuberbier T Bachert C Baena-Cagnani CE Bousquet PJ Development and implementation of guidelines in allergic rhinitis—an ARIA-GA_2_LEN paper. Allergy. (2010) 65:1212–21. 10.1111/j.1398-9995.2010.02439.x 20887423

[2] Di Bona D Plaia A Leto-Barone MS La Piana S Di Lorenzo G. Efficacy of grass pollen allergen sublingual immunotherapy tablets for seasonal allergic rhinoconjunctivitis: a systematic review and meta-analysis. JAMA Intern Med. (2015) 175:1301–9. 10.1001/jamainternmed.2015.2840 26120825

[3] Di Bona D Plaia A Leto-Barone MS La Piana S Di Lorenzo G. Efficacy of subcutaneous and sublingual immunotherapy with grass allergens for seasonal allergic rhinitis: a meta-analysis-based comparison. J Allergy Clin Immunol. (2012) 130:1097–1107.e2. 10.1016/j.jaci.2012.08.012 23021885

[4] Radulovic S Calderon MA Wilson D Durham S. Sublingual immunotherapy for allergic rhinitis. Cochrane Database Syst Rev. (2010) (12):CD002893. 10.1002/14651858.CD002893.pub2 21154351 PMC7001038

[5] Calderon MA Alves B Jacobson M Hurwitz B Sheikh A Durham S. Allergen injection immunotherapy for seasonal allergic rhinitis. Cochrane Database Syst Rev. (2007) (1):CD001936. 10.1002/14651858.CD001936.pub2 17253469 PMC7017974

[6] Dretzke J Meadows A Novielli N Huissoon A Fry-Smith A Meads C. Subcutaneous and sublingual immunotherapy for seasonal allergic rhinitis: a systematic review and indirect comparison. J Allergy Clin Immunol. (2013) 131:1361–6. 10.1016/j.jaci.2013.02.013 23557834

[7] Batard T Dreux S Rouet M Jain K Péguillat C Delecroix M Impact of the standardization unit’s definition on the *in vitro* biological potency of allergen extracts. Explor Asthma Allergy. (2023) 1:107–14. 10.37349/eaa.2023.00012

[8] Batard T Dreux S Jain K Baveux D Péguillat C Villardsaussine S Influence of the definition of the standardization unit on the *in vitro* potency of cat allergen extracts. Explor Asthma Allergy. (2025) 3:100968. 10.37349/eaa.2025.100968

[9] Bachert C Larché M Bonini S Canonica GW Kündig T Larenas-Linnemann D Allergen immunotherapy on the way to product-based evaluation—a WAO statement. World Allergy Organ J. (2015) 8:29. 10.1186/s40413-015-0078-8 26417398 PMC4571059

[10] Page MJ McKenzie JE Bossuyt PM Boutron I Hoffmann TC Mulrow CD The PRISMA 2020 statement: an updated guideline for reporting systematic reviews. Br Med J. (2021) 372:n71. 10.1136/bmj.n71 33782057 PMC8005924

[11] Schünemann H Brożek J Guyatt G Oxman A, editors. GRADE Handbook for Grading Quality of Evidence and Strength of Recommendations. Updated October 2013. Hamilton, ON: The GRADE Working Group (2013). Available at: https://gdt.gradepro.org/app/handbook/handbook.html (Accessed March 06, 2025).

[12] Higgins JPT Thomas J Chandler J Cumpston M Li T Page MJ, editors. Cochrane Handbook for Systematic Reviews of Interventions Version 6.3. London: Cochrane (2022). Available at: https://training.cochrane.org/handbook/archive/v6.3 (Accessed March 06, 2025).

[13] Roberts G Pfaar O Akdis CA Ansotegui IJ Durham SR Gerth van Wijk R EAACI guidelines on allergen immunotherapy: allergic rhinoconjunctivitis. Allergy. (2018) 73:765–98. 10.1111/all.13317 28940458

[14] DerSimonian R Laird N. Meta-analysis in clinical trials. Control Clin Trials. (1986) 7:177–88. 10.1016/0197-2456(86)90046-2 3802833

[15] Li T Higgins JPT Deeks JJ. Chapter 5: Collecting data. In: Higgins JPT Thomas J Chandler J Cumpston M Li T Page MJ Welch VA, editors. Cochrane Handbook for Systematic Reviews of Interventions Version 6.5. London: Cochrane (2024). Available at: www.training.cochrane.org/handbook (Accessed March 06, 2025).

[16] Higgins JPT Savović J Page MJ Elbers RG Sterne JAC. Chapter 8: Assessing risk of bias in a randomized trial. In: Higgins JPT Thomas J Chandler J Cumpston M Li T Page MJ Welch VA, editors. Cochrane Handbook for Systematic Reviews of Interventions Version 6.2. London: Cochrane (2021). Available at: https://training.cochrane.org/handbook/archive/v6.2 (Accessed March 06, 2025).

[17] Higgins JP Thompson SG. Quantifying heterogeneity in a meta-analysis. Stat Med. (2002) 21:1539–58. 10.1002/sim.1186 12111919

[18] Baujat B Mahé C Pignon J-P Hill C. A graphical method for exploring heterogeneity in meta-analyses: application to a meta-analysis of 65 trials. Stat Med. (2002) 21:2641–52. 10.1002/sim.1221 12228882

[19] Egger M Davey Smith G Schneider M Minder C. Bias in meta-analysis detected by a simple, graphical test. Br Med J. (1997) 315:629–34. 10.1136/bmj.315.7109.629 9310563 PMC2127453

[20] GRADEpro GDT: GRADEpro Guideline Development Tool [Software]. McMaster University and Evidence Prime (2021). Available at: https://www.gradepro.org (Accessed March 06, 2025).

[21] Viechtbauer W. Metafor: Meta-Analysis Package for R. R package Version 2010. Available at: http://cran.r-project.org/web/packages/metafor/index.html (Accessed March 06, 2025).

[22] Review Manager (RevMan) [Computer program]: Version 5.0. The Cochrane Collaboration, London, United Kingdom (2012).

[23] ProMeta [Computer software]. Version 2.0. Internovi, Cesena, Italy.

[24] Sabbah A Hassoun S Le Sellin J André C Sicard H. A double-blind, placebo-controlled trial by the sublingual route of immunotherapy with a standardized grass pollen extract. Allergy. (1994) 49:309–13. 10.1111/j.1398-9995.1994.tb02273.x 8092425

[25] Clavel R Bousquet J André C. Clinical efficacy of sublingual-swallow immunotherapy: a double-blind, placebo-controlled trial of a standardized five-grass-pollen extract in rhinitis. Allergy. (1998) 53:493–8. 10.1111/j.1398-9995.1998.tb04086.x 9636808

[26] Ott H Sieber J Brehler R Fölster-Holst R Kapp A Klimek L Efficacy of grass pollen sublingual immunotherapy for three consecutive seasons and after cessation of treatment: the ECRIT study. Allergy. (2009) 64:1394–401. 10.1111/j.1398-9995.2009.02194.x 19764942

[27] Stelmach I Kaczmarek-Woźniak J Majak P Olszowiec-Chlebna M Jerzynska J. Efficacy and safety of high-doses sublingual immunotherapy in ultra-rush scheme in children allergic to grass pollen. Clin Exp Allergy. (2009) 39:401–8. 10.1111/j.1365-2222.2008.03159.x 19134016

[28] Kałuzińska-Parzyszek I Majak P Jerzyńska J Smejda K Stelmach I. Immunoterapia podjęzykowa jest skuteczna i bezpieczna u dzieci. Alergia Astma Immunologia. (2011) 16:139–44.

[29] Stelmach I Kałuzińska-Parzyszek I Jerzynska J Stelmach P Stelmach W Majak P. Comparative effect of pre-coseasonal and continuous grass sublingual immunotherapy in children. Allergy. (2012) 67:312–20. 10.1111/j.1398-9995.2011.02758.x 22142341

[30] Bozek A Kolodziejczyk K Warkocka-Szoltysek B Jarzab J. Grass pollen sublingual immunotherapy: a double-blind, placebo-controlled study in elderly patients with seasonal allergic rhinitis. Am J Rhinol Allergy. (2014) 28:423–7. 10.2500/ajra.2014.28.4091 25198030

[31] Kralimarkova TZ Popov TA Staevska M Mincheva R Lazarova C Racheva R Objective approach for fending off the sublingual immunotherapy placebo effect in subjects with pollenosis: double-blinded, placebo-controlled trial. Ann Allergy Asthma Immunol. (2014) 113:108–13. 10.1016/j.anai.2014.03.019 24745701

[32] Sieber J Neis M Brehler R Fölster-Holst R Kapp A Klimek L Increasing long-term safety of seasonal grass pollen sublingual immunotherapy: the ECRIT study. Expert Opin Drug Saf. (2012) 11:7–13. 10.1517/14740338.2012.626765 21980934

[33] Ahmadiafshar A Maarefvand M Taymourzade B Mazloomzadeh S Torabi Z. Efficacy of sublingual swallow immunotherapy in children with rye grass pollen allergic rhinitis: a double-blind placebo-controlled study. Iran J Allergy Asthma Immunol. (2012) 11:175–81. 22761191

[34] Mungan D Misirligil Z Gürbüz L. Comparison of the efficacy of subcutaneous and sublingual immunotherapy in mite-sensitive patients with rhinitis and asthma–a placebo controlled study. Ann Allergy Asthma Immunol. (1999) 82:485–90. 10.1016/S1081-1206(10)62726-3 10353581

[35] Guez S Vatrinet C Fadel R André C. House-dust-mite sublingual-swallow immunotherapy (SLIT) in perennial rhinitis: a double-blind, placebo-controlled study. Allergy. (2000) 55:369–75. 10.1034/j.1398-9995.2000.00413.x 10782522

[36] Bahçeciler NN Işik U Barlan IB Başaran MM. Efficacy of sublingual immunotherapy in children with asthma and rhinitis: a double-blind, placebo-controlled study. Pediatr Pulmonol. (2001) 32:49–55. 10.1002/ppul.1088 11416876

[37] Tseng SH Fu LS Nong BR Weng JD Shyur SD. Changes in serum specific IgG4 and IgG4/IgE ratio in mite-sensitized Taiwanese children with allergic rhinitis receiving short-term sublingual-swallow immunotherapy: a multicenter, randomized, placebo-controlled trial. Asian Pac J Allergy Immunol. (2008) 26:105–12. 19054928

[38] O'Hehir RE Gardner LM de Leon MP Hales BJ Biondo M Douglass JA House dust mite sublingual immunotherapy: the role for transforming growth factor-beta and functional regulatory T cells. Am J Respir Crit Care Med. (2009) 180:936–47. 10.1164/rccm.200905-0686OC 19696440

[39] Aydogan M Eifan AO Keles S Akkoc T Nursoy MA Bahceciler NN Sublingual immunotherapy in children with allergic rhinoconjunctivitis mono-sensitized to house-dust-mites: a double-blind-placebo-controlled randomised trial. Respir Med. (2013) 107:1322–9. 10.1016/j.rmed.2013.06.021 23886432

[40] Bozek A Ignasiak B Filipowska B Jarzab J. House dust mite sublingual immunotherapy: a double-blind, placebo-controlled study in elderly patients with allergic rhinitis. Clin Exp Allergy. (2013) 43:242–8. 10.1111/cea.12039 23331565

[41] Potter PC Baker S Fenemore B Nurse B. Clinical and cytokine responses to house dust mite sublingual immunotherapy. Ann Allergy Asthma Immunol. (2015) 114:327–34. 10.1016/j.anai.2014.12.015 25661658

[42] Di Rienzo V Pucci S D’Alo S Di Cara G Incorvaia C Frati F Effects of high-dose sublingual immunotherapy on quality of life in patients with cypress-induced rhinitis: a placebo-controlled study. Clin Exp Allergy. (2006) 6:67–70. 10.1111/j.1365-2222.2005.00102.x

[43] Khinchi MS Poulsen LK Carat F André C Hansen AB Malling HJ. Clinical efficacy of sublingual and subcutaneous birch pollen allergen-specific immunotherapy: a randomized, placebo-controlled, double-blind, double-dummy study. Allergy. (2004) 59:45–53. 10.1046/j.1398-9995.2003.00387.x 14674933

[44] Vervloet D Birnbaum J Laurent P Hugues B Fardeau MF Massabie-Bouchat YP Safety and efficacy of Juniperus ashei sublingual-swallow ultra-rush pollen immunotherapy in cypress rhinoconjunctivitis. A double-blind, placebo-controlled study. Int Arch Allergy Immunol. (2007) 142:239–46. 10.1159/000097026 17114889

[45] Voltolini S Troise C Incorvaia C Bignardi D Di Cara G Marcucci F Effectiveness of high dose sublingual immunotherapy to induce a stepdown of seasonal asthma: a pilot study. Curr Med Res Opin. (2010) 26:37–40. 10.1185/03007990903431886 19895362

[46] Vourdas D Syrigou E Potamianou P Carat F Batard T André C Double-blind, placebo-controlled evaluation of sublingual immunotherapy with standardized olive pollen extract in pediatric patients with allergic rhinoconjunctivitis and mild asthma due to olive pollen sensitization. Allergy. (1998) 53:662–72. 10.1111/j.1398-9995.1998.tb03952.x 9700035

[47] Worm M Rak S de Blay F Malling HJ Melac M Cadic V Sustained efficacy and safety of a 300IR daily dose of a sublingual solution of birch pollen allergen extract in adults with allergic rhinoconjunctivitis: results of a double-blind, placebo-controlled study. Clin Transl Allergy. (2014) 4:7. 10.1186/2045-7022-4-7 24517417 PMC3928083

[48] Bowen T Greenbaum J Charbonneau Y Hebert J Filderman R Sussman G Canadian Trial of sublingual swallow immunotherapy for ragweed rhinoconjunctivitis. Ann Allergy Asthma Immunol. (2004) 93:425–30. 10.1016/S1081-1206(10)61408-1 15562880

[49] La Rosa M Ranno C André C Carat F Tosca MA Canonica GW. Double-blind placebo-controlled evaluation of sublingual-swallow immunotherapy with standardized *Parietaria judaica* extract in children with allergic rhinoconjunctivitis. J Allergy Clin Immunol. (1999) 104:425–32. 10.1016/s0091-6749(99)70388-x 10452766

[50] Dhami S Nurmatov U Arasi S Khan T Asaria M Zaman H Allergen immunotherapy for allergic rhinoconjunctivitis: a systematic review and meta-analysis. Allergy. (2017) 72:1597–631. 10.1111/all.13201 28493631

[51] Wang L Yin J Fadel R Montagut A de Beaumont O Devillier P. House dust mite sublingual immunotherapy is safe and appears to be effective in moderate, persistent asthma. Allergy. (2014) 69:1181–8. 10.1111/all.12188 25056584

[52] Thétis-Soulié M Hosotte M Grozelier I Baillez C Scurati S Mercier V. The MaDo real-life study of dose adjustment of allergen immunotherapy liquid formulations in an indication of respiratory allergic disease: reasons, practices, and outcomes. Front Allergy. (2022) 3:971155. 10.3389/falgy.2022.971155 36017469 PMC9395981

[53] Demoly P Molimard M Bergmann JF Delaisi B Gouverneur A Vadel J Impact of liquid sublingual immunotherapy on asthma onset and progression in patients with allergic rhinitis: a nationwide population-based study (EfficAPSI study). Lancet Reg Health Eur. (2024) 41:100915. 10.1016/j.lanepe.2024.100915. Erratum in: *Lancet Reg Health Eur*. (2024) 46:101120. doi: 10.1016/j.lanepe.2024.101120. 38707866 PMC11066575

[54] Penagos M Durham SR. Allergen immunotherapy for long-term tolerance and prevention. J Allergy Clin Immunol. (2022) 149:802–11. 10.1016/j.jaci.2022.01.007 35085663

[55] Trebuchon F David M Demoly P. Medical management and sublingual immunotherapy practices in patients with house dust mite-induced respiratory allergy: a retrospective, observational study. Int J Immunopathol Pharmacol. (2012) 25:193–206. 10.1177/039463201202500122 22507332

[56] Blome C Hadler M Karagiannis E Kisch J Neht C Kressel N Relevant patient benefit of sublingual immunotherapy with birch pollen allergen extract in allergic rhinitis: an open, prospective, non- interventional study. Adv Ther. (2020) 37:2932–45. 10.1007/s12325-020-01345-7 32342352 PMC7467431

[57] Wahn U Bachert C Heinrich J Richter H Zielen S. Real-world benefits of allergen immunotherapy for birch pollen-associated allergic rhinitis and asthma. Allergy. (2019) 74:594–604. 10.1111/all.13598 30183091 PMC6585786

