# Correction: Efficacy of index of reactivity-liquid sublingual immunotherapy in allergic rhinoconjunctivitis: a systematic review and meta-analysis of randomized studies

**DOI:** 10.3389/falgy.2025.1652198

**Published:** 2025-07-25

**Authors:** Danilo Di Bona, Andrea Di Biase, Giovanni Paoletti, Rosanna Villani, Gaetano Serviddio, Josiane Cognet-Sicé, Silvia Scurati, Giorgio Walter Canonica

**Affiliations:** ^1^ Department of Medical and Surgical Sciences, University of Foggia, Foggia, Italy; ^2^ Department of Biomedical Sciences, Humanitas University, Pieve Emanuele, Italy; ^3^ Personalized Medicine, Asthma and Allergy, Humanitas Clinical and Research Center, IRCCS, Rozzano, Italy; ^4^ Integrated Health Care Department, Stallergenes Greer, Antony, France

**Keywords:** meta-analysis, randomized controlled trial, rhinitis, allergic, SLIT-liquid, sublingual immunotherapy, systematic review

In section Methods, subsection Data analysis, paragraph 2: the reference for “In cases where standard errors (SEs) were provided, SDs were calculated using the formula: *SD* = *SE*√*n*” was erroneously written as (13). It should be (15) as stated below.

“In cases where standard errors (SEs) were provided, SDs were calculated using the formula: *SD* = *SE*√*n* (15).”

In section Results, paragraph 3: the references for “Low heterogeneity was observed (Q = 0.37; df = 25; *P* = 0.22; I² = 20%) but decreased to 0% after excluding three outlier studies” were erroneously written as (26, 29, 42). It should be (28, 31, 44) as stated below.

“Low heterogeneity was observed (Q = 0.37; df = 25; *P* = 0.22; I² = 20%) but decreased to 0% after excluding three outlier studies (28, 31, 44)”

In section Results, paragraph 6: the references for “However, excluding four influential studies” were erroneously written as (26, 27, 31, 32). It should be (26, 28, 29, 34) as stated below.

“However, excluding four influential studies (26, 28, 29, 34)…”

There was a mistake in the caption of Figure 1 as published. The term “seasonal” was incorrectly inserted in the sentence “Meta-analysis of 25 RCTs of IR-SLIT-liquid vs. placebo for seasonal allergic rhinoconjunctivitis.” and should be deleted. The corrected caption of Figure 1 appears below.

“Meta-analysis of 25 RCTs of IR-SLIT-liquid vs. placebo for allergic rhinoconjunctivitis. The SMD and 95% CI for the effect of treatment on symptom score (SS) are plotted on the graph.”

There was a mistake in the caption of Figure 3 as published. The term “seasonal” was incorrectly inserted in the sentence “Meta-analysis of 20 RCTs of IR-SLIT-liquid vs. placebo for seasonal allergic rhinoconjunctivitis.” and should be deleted. The corrected caption of Figure 3 appears below.

“Meta-analysis of 20 RCTs of IR-SLIT-liquid vs. placebo for allergic rhinoconjunctivitis. The SMD and 95% CI for the effect of treatment on medication score (MS) are plotted on the graph.”

The original version of this article has been updated.

