# Supplementary material for: Efficacy of index of reactivity-liquid sublingual immunotherapy in allergic rhinoconjunctivitis: a systematic review and meta-analysis of randomized studies
Source: Front Allergy. 2025 Jun 5;6:1597003. doi: 10.3389/falgy.2025.1597003 (PMC12176734; doi:10.3389/falgy.2025.1597003)
Supplement: Supplementary file 1 [file Datasheet1.docx]

Supplementary Material

# Supplementary Tables

**Supplementary Table 1.** Search strategy

#1: sublingual immunotherapy grass AND (randomizedcontrolledtrial[Filter])

#2: sublingual immunotherapy house dust mite AND (randomizedcontrolledtrial[Filter]

#3: sublingual immunotherapy cypress AND (randomizedcontrolledtrial[Filter]

#4: sublingual immunotherapy juniper AND (randomizedcontrolledtrial[Filter])

#5: sublingual immunotherapy ragweed AND (randomizedcontrolledtrial[Filter]

#6: sublingual immunotherapy olive AND (randomizedcontrolledtrial[Filter]

#7: sublingual immunotherapy parietaria AND (randomizedcontrolledtrial[Filter]

#8: sublingual immunotherapy pellitory AND (randomizedcontrolledtrial[Filter]

#1 OR #2 OR #3 OR #4 OR #5 OR #6 OR #7 OR #8

**Supplementary Table 2.** Sensitivity analysis for symptom and medication scores

| **Symptom Score** | | **n** | | **Point estimate**  **(95%CI)** | | ***I^2^*** | |
| --- | --- | --- | --- | --- | --- | --- | --- |
| Available data  Estimated data | | 1,314  369 | | -0.34 (-0.45, -0.23)  -0.32 (-0.54, -0.09) | | 0%  35% | |
| Low RoB  High RoB/some concerns | | 1,095  735 | | -0.45 (-0.61, -0.29)  -0.15 (-0.30, -0.00) | | 21%  0% | |
| Sample size ≥56  Sample size <56 | | 1,476  354 | | -0.29 (-0.42, -0.16)  -0.33 (-0.56, -0.10) | | 27%  13% | |
| Excluding influential studies  (Khinchi, Kralimarkova, Vervloet) | | 1,678 | | -0.35 (-0.45, -0.26) | | 0% | |
| Excluding studies with duplicated controls (Stelmach 2012) | | 1,758 | | -0.29 (-0.42, -0.17) | | 24% | |
| **Medication Score** | **n** | | **Point estimate**  **(95%CI)** | | ***I^2^*** | |  |
| Available data  Estimated data | 1,331  244 | | -0.40 (-0.61, -0.19)  -0.88 (-1.55, -0.21) | | 63%  81% | |  |
| Low RoB  High RoB/some concerns | 1,003  552 | | -0.65 (-1.02, -0.27)  -0.40 (-0.66, -0.15) | | 82%  49% | |  |
| Sample size ≥56  Sample size <56 | 1,248  307 | | -0.33 (-0.48, -0.17)  -0.85 (-1.38, 0.31) | | 35%  79% | |  |
| Excluding influential studies  (Ott, Stelmach 2012, Mungan, Kaluzinska) | 1,158 | | -0.42 (-0.54, -0.31) | | 0% | |  |
| Excluding studies with duplicated controls (Stelmach 2012) | 1,483 | | -0.47 (-0.68, -0.27) | | 65% | |  |

REM, random effects model; FEM, fixed effects model; CI, confidence interval

**Supplementary Table 3.** Evidence summary

| **Certainty assessment** | | | | | | | **№ of patients** | | **Effect** | | **Certainty** | **Importance** |
| --- | --- | --- | --- | --- | --- | --- | --- | --- | --- | --- | --- | --- |
| **№ of studies** | **Study design** | **Risk of bias** | **Inconsistency** | **Indirectness** | **Imprecision** | **Other considerations** | **SLIT** | **PLACEBO** | **Relative (95% CI)** | **Absolute (95% CI)** |  |  |
| **SYMPTOM SCORE (follow-up: range 3 months to 36 months)** | | | | | | | | | | | | |
| 25 | randomised trials | not serious | serious^a^ | not serious | not serious | none | 962 | 868 | - | SMD **0.3 SD lower** (0.41 lower to 0.18 lower) | ⨁⨁⨁◯ Moderate | IMPORTANT |
| **MEDICATION SCORE (follow-up: range 3 months to 36 months)** | | | | | | | | | | | | |
| 20 | randomised trials | not serious | serious^a^ | not serious | not serious | none | 814 | 741 | - | SMD **0.51 SD lower** (0.72 lower to 0.29 lower) | ⨁⨁⨁◯ Moderate | IMPORTANT |

CI, confidence interval; SMD, standardised mean difference

a. 95% CI of some studies do not overlap

.

# Supplementary Figures


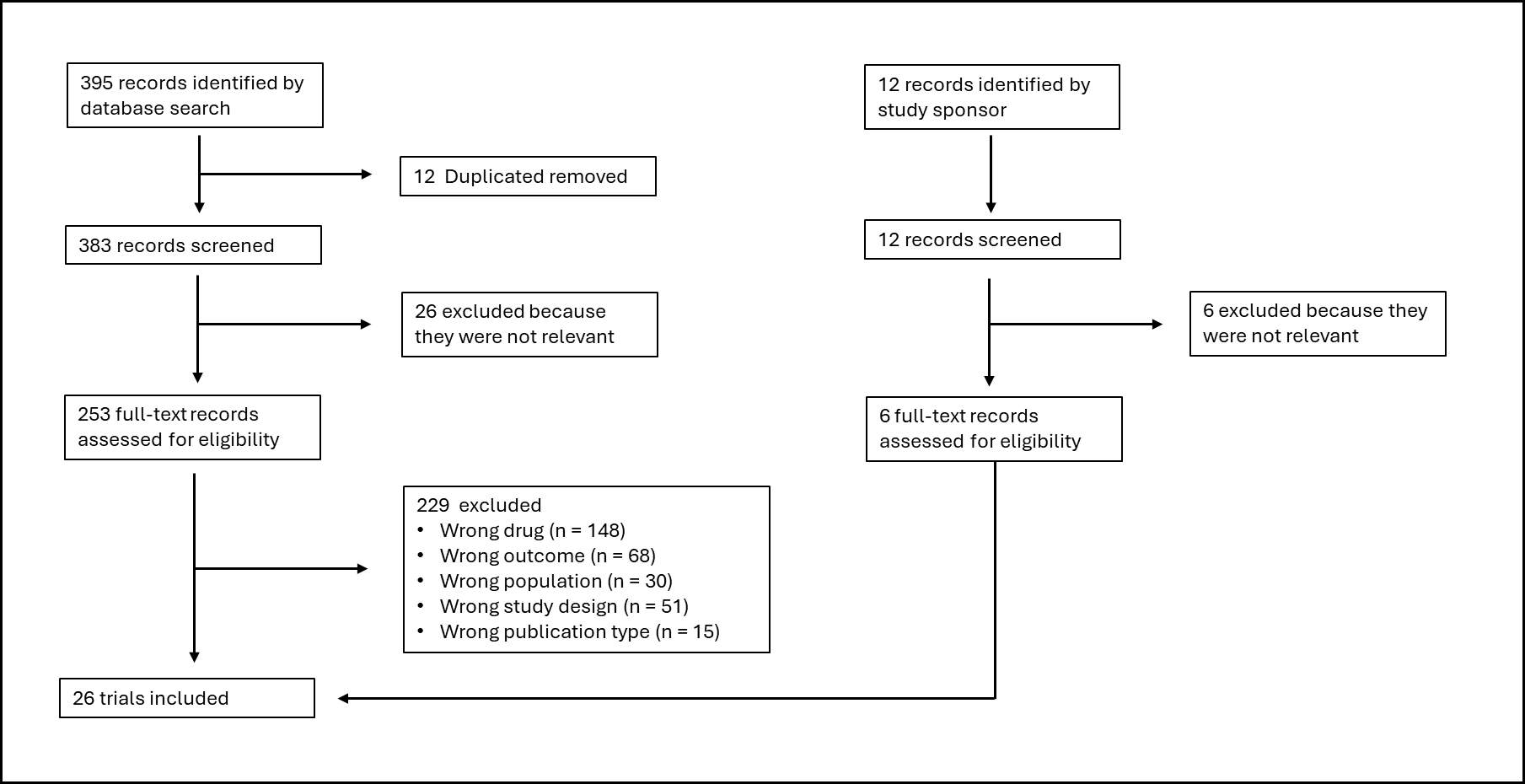


**Supplementary Figure 1.** Study selection


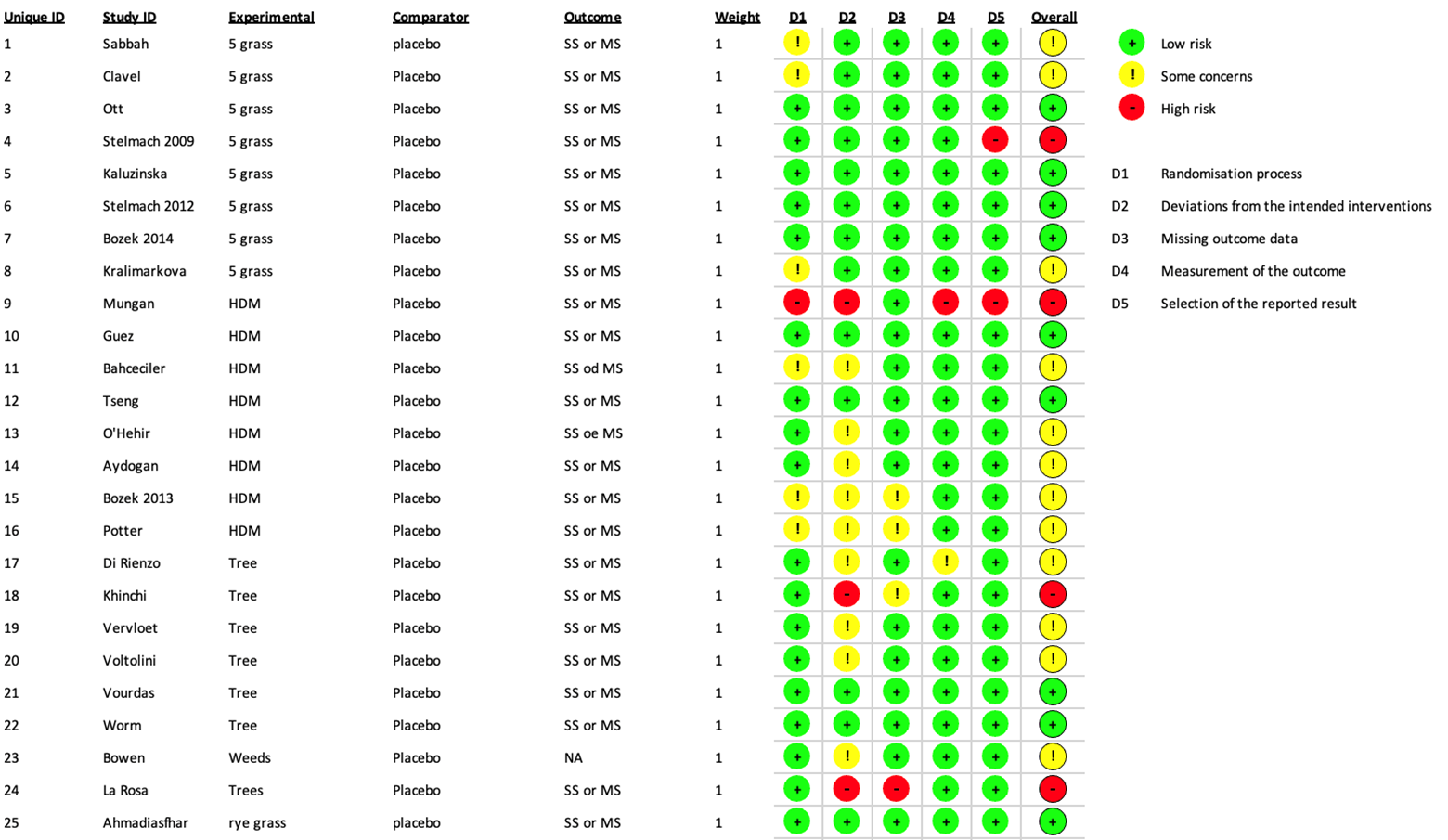


**Supplementary Figure 2.** Risk of bias of RCTs included.


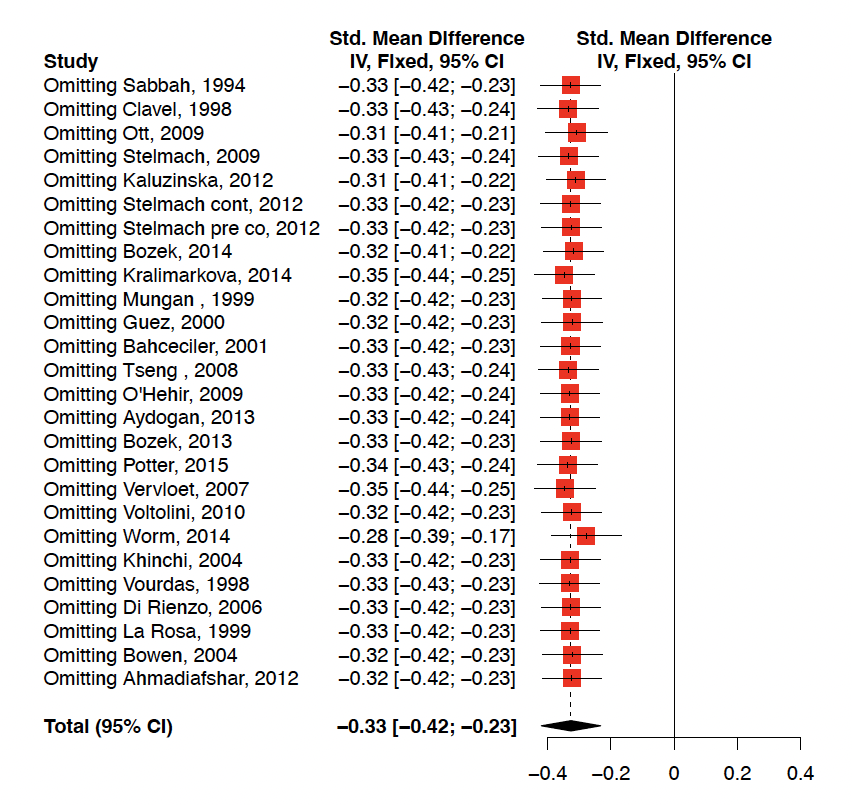

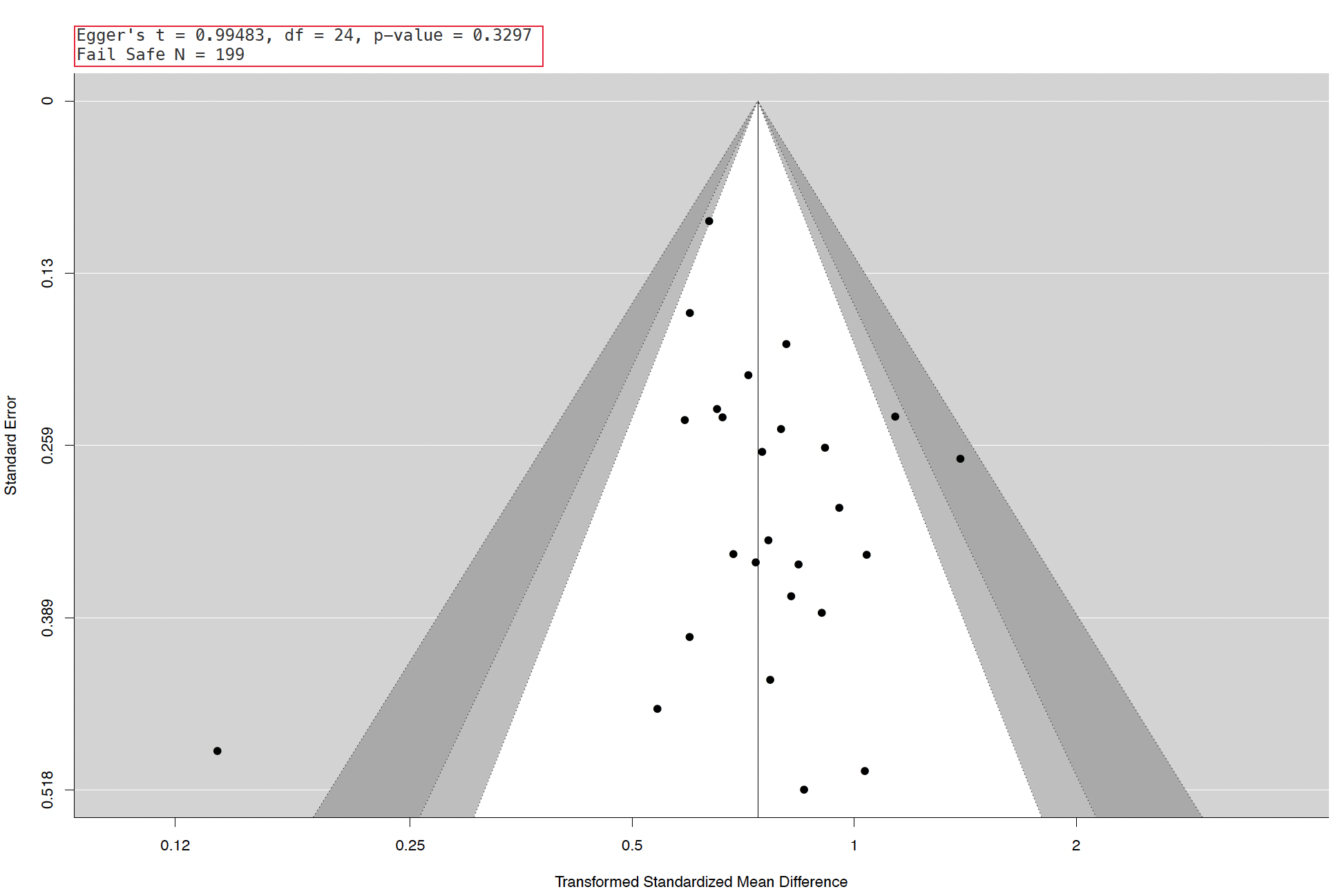


**C**

**B**

**A**


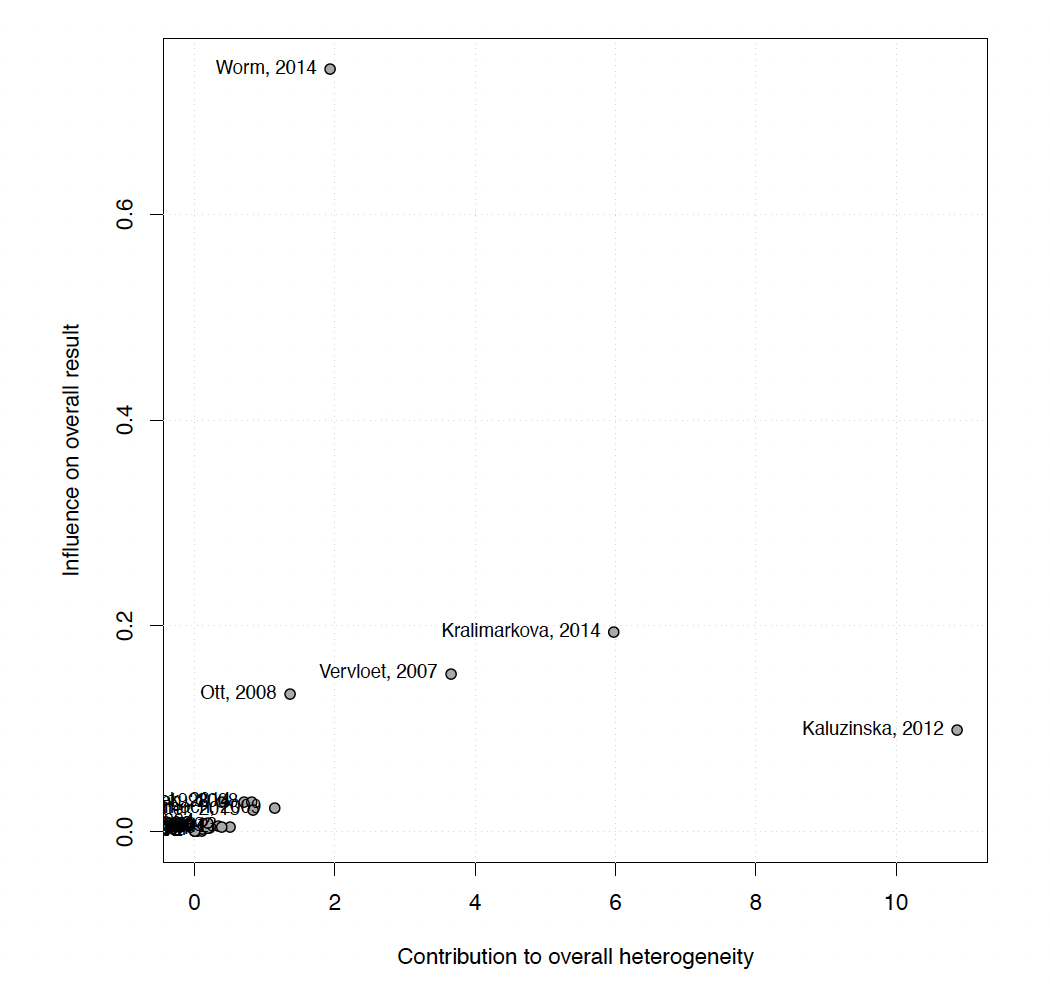


**Supplementary Figure 3.** Funnel plot (A), analysis of outliers (B) and sensitivity analysis (C) for SS.


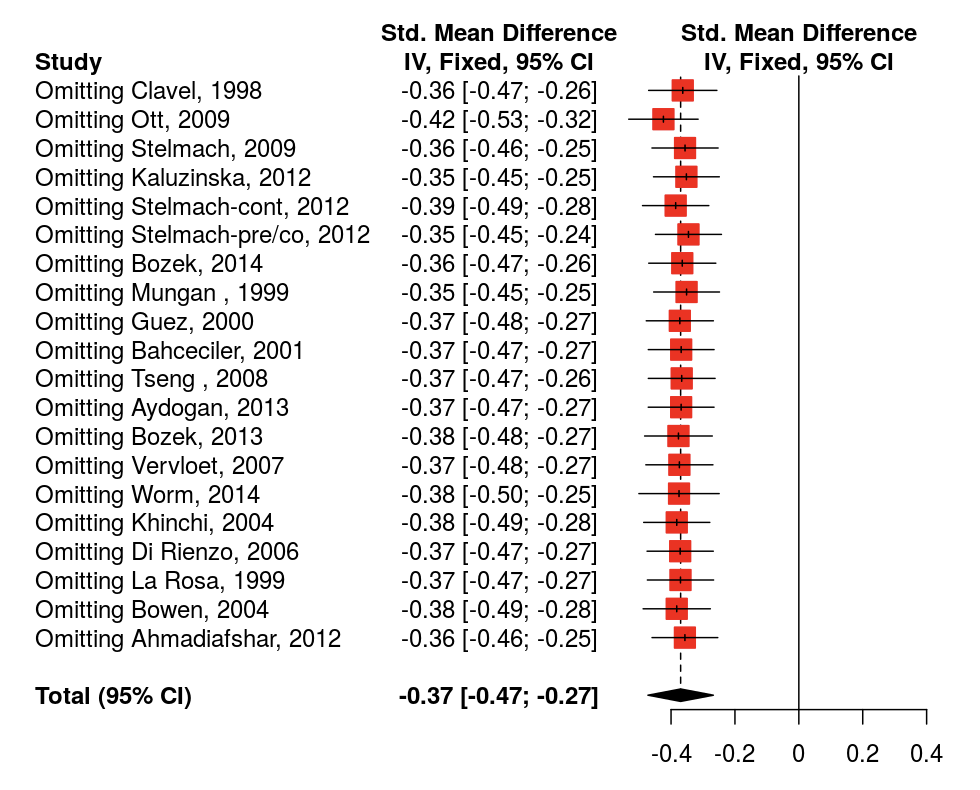

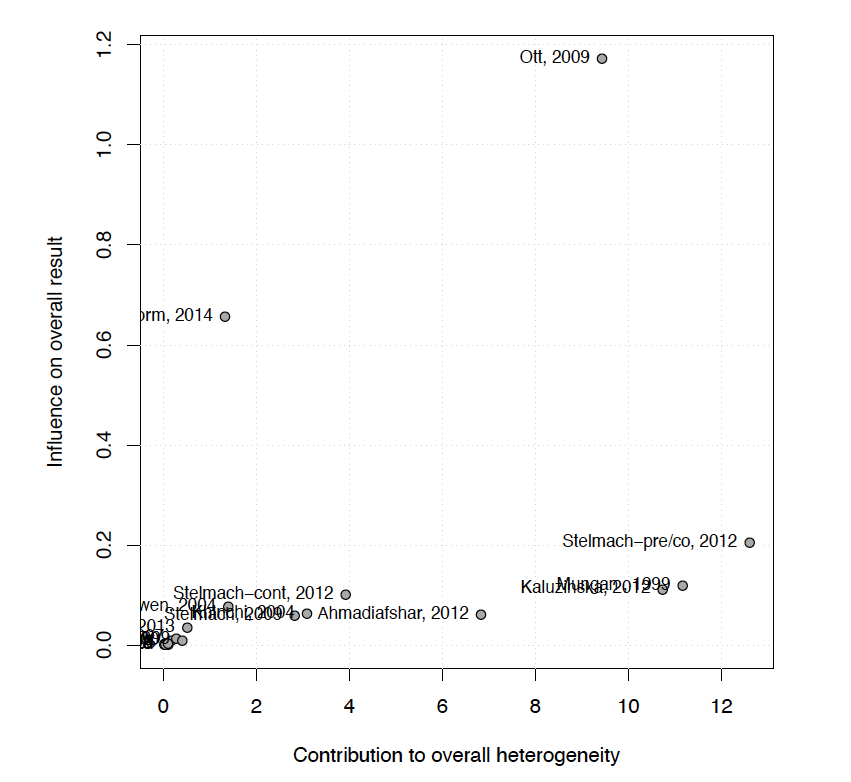

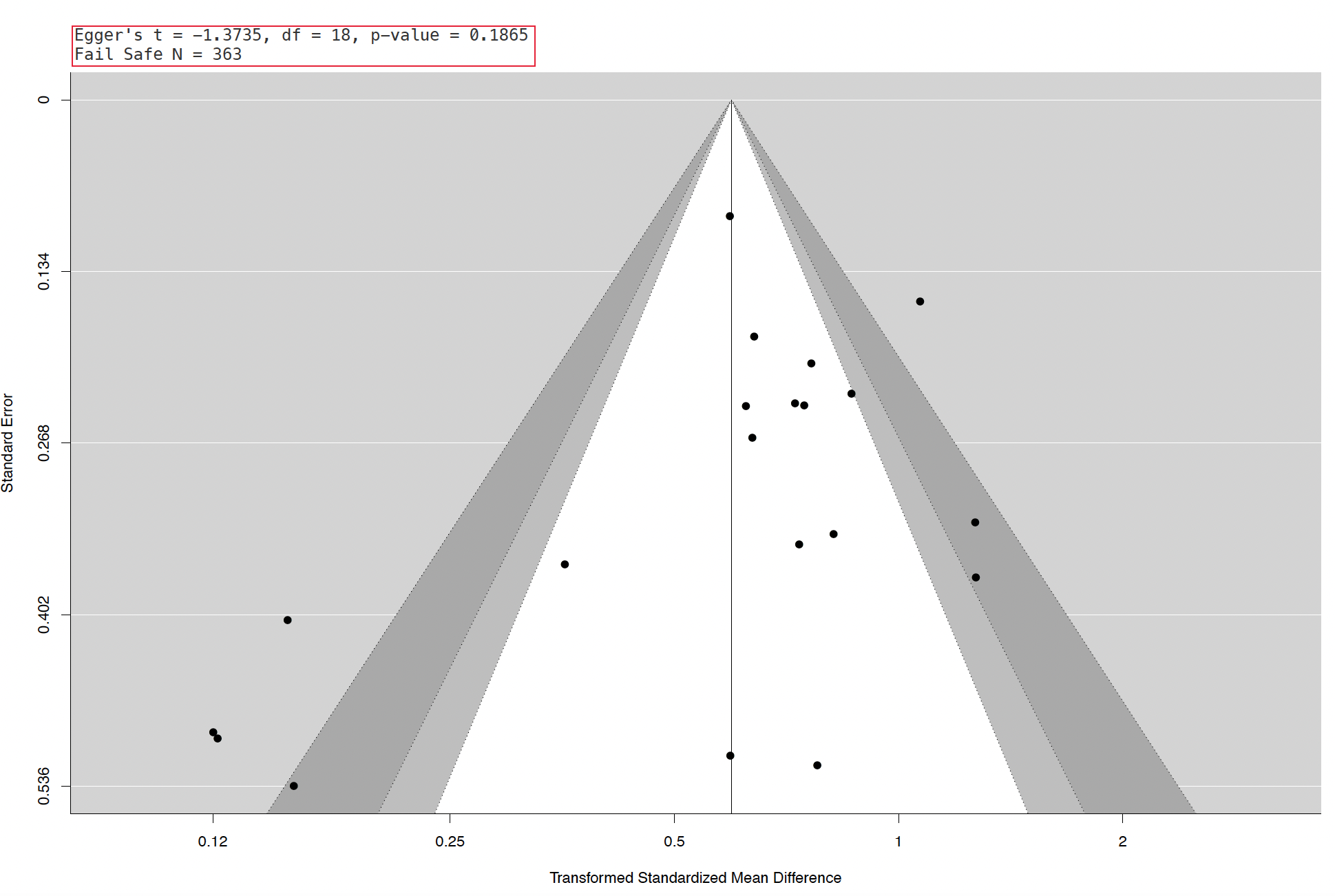


**C**

**Supplementary Figure 4.** Funnel plot (A), analysis of outliers (B) and sensitivity analysis (C) for MS.

**B**

**A**

C
